# Phenolic Compounds in *Brassica* Vegetables

**DOI:** 10.3390/molecules16010251

**Published:** 2010-12-30

**Authors:** María Elena Cartea, Marta Francisco, Pilar Soengas, Pablo Velasco

**Affiliations:** Misión Biológica de Galicia, Consejo Superior de Investigaciones Científicas (CSIC), Apartado 28, 36080 Pontevedra, Spain; Email: mfrancisco@mbg.cesga.es (M.F.); psoengas@mbg.cesga.es (P.S.); pvelasco@mbg.cesga.es (P.V.)

**Keywords:** anthocyanins, antioxidant activity, biological activity, brassica, flavonoids, health, hydroxycinnamic acids, polyphenols

## Abstract

Phenolic compounds are a large group of phytochemicals widespread in the plant kingdom. Depending on their structure they can be classified into simple phenols, phenolic acids, hydroxycinnamic acid derivatives and flavonoids. Phenolic compounds have received considerable attention for being potentially protective factors against cancer and heart diseases, in part because of their potent antioxidative properties and their ubiquity in a wide range of commonly consumed foods of plant origin. The *Brassicaceae* family includes a wide range of horticultural crops, some of them with economic significance and extensively used in the diet throughout the world. The phenolic composition of *Brassica* vegetables has been recently investigated and, nowadays, the profile of different *Brassica* species is well established. Here, we review the significance of phenolic compounds as a source of beneficial compounds for human health and the influence of environmental conditions and processing mechanisms on the phenolic composition of *Brassica* vegetables.

## 1. Introduction

Plant-based foods contain significant amounts of bioactive compounds, which provide desirable health benefits beyond basic nutrition. Epidemiological evidence suggests that consumption of a diet rich in vegetables and fruits has positive implications for human health. In the last decades, special attention has been paid towards edible plants, especially those that are rich in secondary metabolites (frequently called phytochemicals) and nowadays, there is an increasing interest in the antioxidant activity of such phytochemicals present in diet. Recent reports suggest that cruciferous vegetables act as a good source of natural antioxidants due to the high levels of carotenoids, tocopherols and ascorbic acid, and strong epidemiological evidence shows that these compounds may help to protect the human body against damage by reactive oxygen species. In addition to carotenoids, tocopherols, and ascorbic acid, most of the antioxidative effect related to plant food intake is mainly due to the presence of phenolic compounds, which have been associated with flavour and colour characteristics of fruits and vegetables. In this aspect, the popularity and consumption of vegetable *Brassica* species is increasing because of their nutritional value. *Brassica* crops have been related to the reduction of the risk of chronic diseases including cardiovascular diseases and cancer. *Brassica* foods are very nutritive, providing nutrients and health-promoting phytochemicals such as vitamins, carotenoids, fiber, soluble sugars, minerals, glucosinolates and phenolic compounds [1,2].

The family *Brassicaceae* (=*Cruciferae*) consists of 350 genera and about 3,500 species, and includes several genera like *Camelina, Crambe, Sinapis, Thlaspi* and *Brassica.* The genus *Brassica* is the most important one within the tribe Brassiceae, which includes some crops and species of great worldwide economic importance such as *Brassica oleracea* L., *Brassica napus* L. and *Brassica rapa* L. The same species can be utilized for several uses according to different forms or types. The genus is categorized into oilseed, forage, condiment, and vegetable crops by using their buds, inflorescences, leaves, roots, seeds, and stems. *Brassicaceae* vegetables represent an important part of the human diet worldwide, are consumed by people all over the world and are considered important food crops in China, Japan, India, and European countries. The main vegetable species is *B. oleracea*, which includes vegetable and forage forms, such as kale, cabbage, broccoli, Brussels sprouts, cauliflower and others; *B. rapa* includes vegetable forms, such as turnip, Chinese cabbage and pak choi, along with forage and oilseed types; *B. napus* crops are mainly used like oilseed (rapeseed), although forage and vegetable types like leaf rape and nabicol are also included; finally, the mustard group which is formed by three species, *B. carinata*, *B. nigra* and *B. juncea*, is mainly used as a condiment although leaves of *B. juncea* are also consumed as vegetables and they are widely used for both fresh and processed markets in Asian countries (Table 1).

## 2. Phenolic Compounds

The beneficial effects of *Brassica* vegetables on health improvement have been partly attributed to their complex mixture of phytochemicals possessing antioxidant activity. In recent years, considerable attention has been directed towards the identification of natural antioxidants, namely those plant-derived that may be used for human consumption regarding health promotion and disease prevention. Among phytochemicals possessing antioxidant capacity, phenolic compounds are one of the most important groups [2]. “Phenolic compounds” is a generic term that refers to a large number of compounds (more than 8,000) widely dispersed throughout the plant kingdom and characterized by having at least one aromatic ring with one or more hydroxyl groups attached. Phenolics are produced in plants as secondary metabolites via the shikimic acid pathway. Phenylalanine ammonialyase (PAL) is the key enzyme catalyzing the biosynthesis of phenolics from the aromatic amino acid phenylalanine.

**Table 1:** Main vegetable *Brassica* species, crops, and plant parts used for consumption.

Species	Group	Common name	Organ
*Brassica oleracea*	*acephala*	Kale, collards	Leaves
	*capitata capitata*	Cabbage	Terminal leaf buds (heads)
	*capitata sabauda*	Savoy cabbage	Terminal leaf buds (heads)
	*costata*	Tronchuda cabbage	Loose heads
	*gemmifera*	Brussels sprouts	Vegetative buds
	*botrytis botrytis*	Cauliflower	Inflorescences
	*botrytis italica*	Broccoli	Inflorescences
	*gongylodes*	Kohlrabi	Stem
	*albogabra*	Chinese kale	Leaves
*Brassica rapa*	*chinensis*	Pak choi, bok choy	Leaves
	*dichotoma*	Brown sarson, toria	Seeds
	*narinosa*	Chinese flat cabbage, wutacai	Leaves
	*nipposinica*	Mibuna, mizuna	Leaves
	*oleifera*	Turnip rape, rapeseed	Seeds
	*pekinensis*	Chinese cabbage, pe-tsai	Leaves
	*perviridis*	Komatsuna, Tendergreen	Leaves
	*parachinensis*	Choy sum	Leaves
	*rapa*	Turnip, turnip greens, turnip tops	Roots, leaves and shoots
	*ruvo*	Broccoleto	Shoots
	*trilochularis*	Yellow sarson	Seeds
*Brassica napus*	*pabularia*	Leaf rape, nabicol	Leaves
	*napobrassica*	Swede, rutabaga	Roots
*Brassica juncea*	*rugosa*	Mustard greens	Leaves
	*capitata*	Head mustard	Heads
	*crispifolia*	Cut leaf mustard	Leaves

Phenolics range from simple, low molecular-weight, single aromatic-ringed compounds to large and complex tannins and derived polyphenols [3,4]. They can be classified based on the number and arrangement of their carbon atoms in flavonoids (flavonols, flavones, flavan-3-ols, anthocyanidins, flavanones, isoflavones and others) and non-flavonoids (phenolic acids, hydroxycinnamates, stilbenes and others) [3] and they are commonly found conjugated to sugars and organic acids. The most widespread and diverse group of polyphenols in *Brassica* species are the flavonoids (mainly flavonols but also anthocyanins) and the hydroxycinnamic acids.

### 2.1. Flavonoids

**Figure 1:**
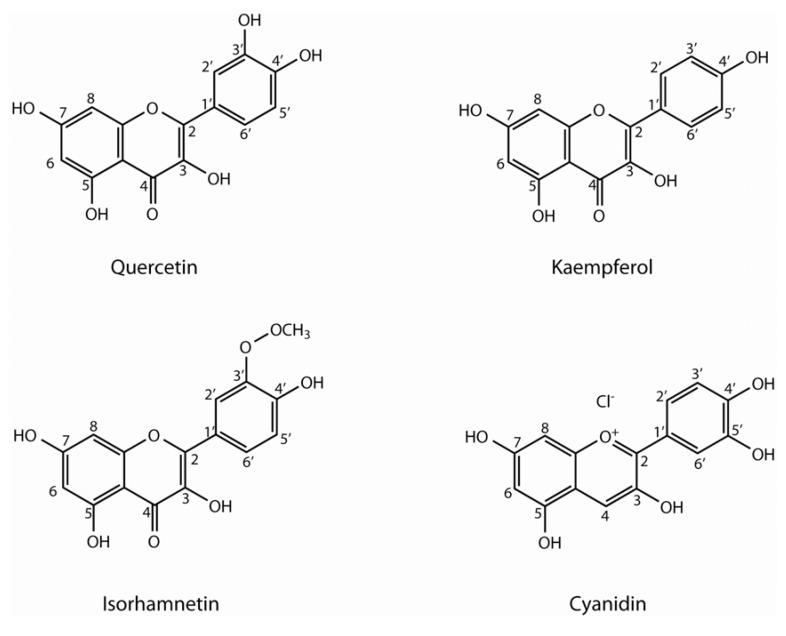
Flavonoid aglycones found in vegetable *Brassica* crops.

Flavonoids are polyphenolic compounds comprising fifteen carbons with two aromatic rings connected by a three-carbon bridge, hence C6-C3-C6 (Figure 1). They are the most numerous of the phenolics and are found throughout the plant kingdom [3,4]. They are present in high concentrations in the epidermis of leaves and fruits and have important and varied roles as secondary metabolites, being involved in processes like UV protection, pigmentation, stimulation of nitrogen-fixing nodules and disease resistance [3,4]. Flavonols are the most widespread of the flavonoids. Quercetin, kaempferol and isorhamnetin, the main flavonols in *Brassica* crops, are most commonly found as *O*-glycosides. Conjugation occurs most frequently at the 3 position of the C-ring, but substitutions can also occur at the 5, 7, 4´, 3´ and 5´ positions [3,5,6]. The number of sugar conjugates is elevated but in *Brassica* vegetables they appeared mainly conjugated to glucose. They are also commonly found acylated by different hydroxycinnamic acids.

Within the colored flavonoids, anthocyanins are the most important group of plant pigments, also considered as multifunctional components of food due to their antioxidant activity and other beneficial biological properties [7,8,9]. Anthocyanins are the sugar-conjugated forms of anthocyanidins, which are compounds widely dispersed throughout the plant kingdom. They are particularly evident in fruit and flower tissues where they are responsible for red, blue and purple colors. In addition, they are also found in leaves, stems, seeds and root tissue. Nevertheless, in certain fruits and vegetables, anthocyanins exist in smaller amounts and only some of them exist in such an amount that they can determine the proper color. They are involved in the protection of plants against excessive light and also have an important role in attracting pollinating insects [3,10]. The chemical structure of the anthocyanin determines the stability, color intensity and potential biological activity. The most common anthocyanins are pelargonidin, cyanidin, delphinidin, peonidin, petunidin and malvidin, being cyanidin the most common in *Brassica* crops [9,11,12]. 

### 2.2. Hydroxycinnamic acids

**Figure 2:**
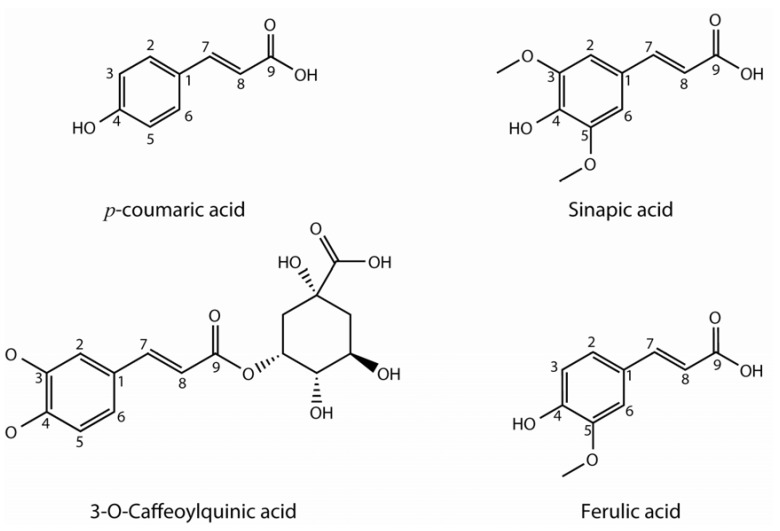
Hydroxycinnamic acids found in vegetable *Brassica* crops.

Hydroxycinnamic acids are a kind of non-flavonoid phenolics characterized by the C6-C3 structure (Figure 2). These compounds are abundant in plants and are used in both structural and chemical plant defense strategies. They can occur freely or as components of plant polymers (cell wall). Derivatives of cinnamic acid are present in numerous vegetables and fruits. In *Brassica* vegetables the most common are *p*-coumaric, sinapic and ferulic acids, often found in conjugation with sugar or other hydroxycinnamic acids [13,14,15,16].

### 2.3. Biological activities

In the past two decades, there has been an increasing interest in the bioavailability and biological effects of phenolics and flavonoids in food plants. Phenolic compounds have multiple additional roles in plants, including attracting insects for seed dispersion and pollination. They are also part of the natural defense system against insects, fungi, viruses and bacteria and they can act as plant hormone controllers. Moreover, in recent years, phenolic compounds have been intensively investigated because of their potential health-promoting effects [2,17,18,19]. They have been reported to possess many useful properties for human health, including anti-inflammatory, enzyme inhibition, antimicrobial, antiallergic, vascular and cytotoxic antitumor activity, but the most important action of phenolics is their antioxidant activity [1,19,20,21,22,23,24]. Furthermore, phenolic compounds possess other properties such as hydrogen peroxide production in the presence of certain metals, the ability to scavenge electrophiles and inhibit nitrosation reactions and chelate metals and, therefore, they act by blocking the initiation of several human diseases [4,25,26,27]. 

The antioxidant activity of phenolic compounds is related with its chemical structure that confers them redox properties. They can play an important role in adsorbing and neutralizing reactive oxygen species (ROS), quenching singlet and triplet oxygen, or decomposing peroxides. Reactive oxygen species, derived from oxidation processes, are an important part of the defense mechanisms against infection, but excessive generation of free oxygen radicals may damage the tissue. When there is an imbalance between ROS and antioxidant defense mechanisms, the ROS lead to the oxidative modification in cellular membranes or intracellular molecules and result in the peroxidation of membrane lipids, leading to the accumulation of lipid peroxides. This oxidative stress has been linked to cancer, aging, atherosclerosis, inflammation and neurodegenerative diseases such as Parkinson’s (PA) and Alzheimer’s disease (AD) [28,29]. Therefore, antioxidants, such as phenolic compounds, are considered as possible protective agents, reducing the oxidative damage from ROS in the human body and retarding the progress of many chronic diseases as well as the oxidation of low-density lipoproteins (LDL), which is thought to play an important role in atherosclerosis. 

The antioxidant ability of flavonoids and phenolic acids is related to the number and position of hydroxyl groups in the molecule; an increase in the number of hydroxyl groups leads to a higher antioxidant activity. Compounds with three hydroxyl groups on the phenyl ring of phenolic acids or the B ring of flavonoids have a high antioxidant activity. The loss of one hydroxyl group decreases activity slightly, whereas the loss of two hydroxyl groups significantly decreases the activity. Moreover, glycosylation results in a lower antioxidant activity for some flavonoids such as quercetin, the addition of a sugar moiety decreases the activity of the aglycon and the addition of a second moiety further decreases the activity, probably due to steric hindrance by addition of sugar moieties [24]. The antioxidant capacity of *Brassica* species has been related to its phenolic profile and content, especially flavonoids, since phenolic compounds have demonstrated a higher antioxidant activity than vitamins and carotenoids [1,20,22,30]. Many studies have shown the antioxidant power of particular flavonoids and flavonoid-rich extracts. Flavonoids can also inhibit, and sometimes induce, a large variety of mammalian enzyme systems; some of these enzymes are involved in important pathways that regulate cell division and proliferation, platelet aggregation, detoxification and inflammatory and immune response. Over the last 15 years, numerous publications have demonstrated that besides the *in vitro* antioxidant capacity, certain phenolic compounds such as anthocyanins, catechins, proanthocyanidins and other non-colored flavonoids may regulate different signaling pathways involved in cell survival, growth and differentiation [1,18,26]. The effects of flavonoids on various stages of the cancer process, on the immune system and on homeostasis in cell systems and animals have been also described [6,27]. However, nowadays, it is widely accepted that if flavonoids have any preventive or curative activity through their ingestion, this effect must involve, not only their antioxidant potential, but also the modulation of multiple cellular pathways that are crucial in the pathogenesis of those diseases [19,25].

Flavonoids are involved in a vast array of biological functions. Quercetin, a major representative of the flavonol subclass and which is found at high concentration in broccoli, has received considerable attention. This flavonoid has displayed the ability to prevent the oxidation of LDL by scavenging free radicals and chelating transition metal ions. These properties are conferred due to the dihydroxylated B-ring, unsaturation at the C-ring and a 4-oxo function at the C-ring. As a result, quercetin may aid in the prevention of certain diseases, such as cancer, atherosclerosis and chronic inflammation by retarding oxidative degradation and inducing enzymes that detoxify carcinogens and also blocking the formation of cancer by deactivating at least 30 types of agents that may cause cancer [26,27,31]. Some flavonoids, derived from quercetin or 3-methoxyquercetin have also been described in literature as acetylcholinesterase (AChE) inhibitors [32]. In addition, kaempferol also revealed a strong antioxidant potential [31]. Some kaempferol derivatives found in high quantities in *Brassica* species are acylated with caffeic acid. The presence of an *O*-dihydroxy structure in the caffeoylmoiety confers great stability to their radical scavenging capacity [33]. Higher intakes of kaempferol resulted in a lower risk of coronary heart disease. It has been demonstrated recently that quercetin and kaempferol synergistically suppress cell proliferation in human gut cancer lines [26]. Ayaz *et al*. [34] found that phenolic fractions extracted from kale leaves (*B. oleracea*), rich in quercetin and kaempferol derivatives, effectively inhibited the growth of the Gram-positive bacteria *Staphylococcus aureus*, *Enterobacter faecalis*, *Bacillus subtilis* and the Gram-negative bacterium *Moraxella catarrhalis*, which is known to be a major respiratory pathogen in humans. Furthermore, isorhamnetin isolated from mustard leaf showed a strong activity in reducing serum levels of glucose in *Diabetes mellitus* through an antioxidant activity test [35]. Isorhamnetin revealed distinct vasodilator effects in animal models as well, suggesting vascular protective effects in human cardiovascular diseases [17,19,36]. Besides, quercetin, kaempferol and isorhamnetin were shown to have an anti-inflammatory effect on activated macrophages [36].

Other phenolic compounds as sinapoyl esters and proanthocyanidins (condensed tannins) are considered undesirable compounds in human nutrition [37,38]. Their presence in oilseed rape meal has antinutritive effects in both monogastric and ruminant livestock feeds [38]. They have been shown to cause a dark colour and a bitter taste in rapeseed meal and derived protein products and, therefore, they are one of the principal factors currently limiting the use of canola seeds (*Brassica napus* L.) and other oilseed crops of the genus *Brassica* including *B. rapa*, *B. juncea* or *B. carinata* [37,38]. Indeed, proanthocyanidins are able to form soluble and insoluble complexes with proteins, polysaccharides and other macromolecules from the diet, thus reducing their bioavailability [38]. Both compounds are abundant in developing seeds of *Brassica* oilseed crops but this paper will focus on *Brassica* vegetables.

### 2.4. Bioavailability

The extent of the absorption of dietary phenolics is an important unsolved problem in judging their potential health effects. The health effects of polyphenols depend on the amount consumed and on their bioavailability. In the last decades, numerous studies have been carried out to determine the bioavailability of different phenolic compounds in diet by using animal models and human assays [39,40,41]. These studies have stated that, in general, most of the phenolic compounds have a low bioavailability since they are detected in very small amounts both in plasma and tissues.

It is well known that the structural properties of polyphenols affect the rate and extent of their absorption in the small intestine and colon of humans, as well as the formation and occurrence of metabolites in plasma [17,40]. Moreover, the absorption, physiological functions and bioavailability of cell wall-bound phenolic compounds differ from those of free phenolic compounds. Phenolic compounds are present as free, as well as cell wall-bound compounds in plants. Bioavailability of flavonoid glycosides takes place in the colon by means of intestinal microflora [41,42].

Flavonoids are thought to be poorly absorbed because the naturally occurring glycosides sugar moieties elevate the hydrophilicity of molecules, and no enzyme is known to split the glycosidic bond. Aglycones, sugar-free flavonoids can efficiently pass through the gut wall, but flavonoids are rarely found as aglycones in plants. It has been suggested that the colon has intestinal microflora that can hydrolyze the glycosidic bond, thus creating the aglycone, but the process also degrades the compound. The aglycone is then absorbed in the large intestine easily because of its lipophilicity, and then metabolized in the liver [42,43]. Bioavailability also differs greatly from one polyphenol to another, so that the most abundant polyphenols in our diet are not necessarily those leading to the highest concentrations of active metabolites in target tissues [44]. To date, many studies analyzing the bioavailability of flavonoids have shown contradictory results, suggesting that the absorption of flavonoids depends on the variety and position of the sugar groups attached. Therefore, research is needed on the bioavailability of phenolics to allow us to correlate phenolic intake with one or several accurate measures of bioavailability (such as concentrations of key bioactive metabolites in plasma and tissues) and with potential health effects in epidemiological studies [41,45]. 

Most of the information on subsequent metabolism is derived from animal studies but little data in humans is available. The limited source of knowledge on the absorption and metabolism of flavonoids has been generated by studying isolated flavonoids and individual foods. More studies are needed on the investigation of the absorption and metabolism of various flavonoids in individual and combinations of foods. 

## 3. Phenolic Compounds in *Brassica* Vegetables

As mentioned above, the nutritional interest of *Brassica* crops is partly related to their phenolic compound contents. These crops are generally rich in polyphenols, but the phenolic compound composition can be quite different among species and even among crops from the same species. The polyphenol composition of different *Brassica* species has been described, revealing distinct qualitative and quantitative profiles. For example, Podsedek [1] did an extensive review on phenolic profiles in different *Brassica* species. Several authors have studied the antioxidant capacity of different cruciferous vegetables and, in general, they found that red cabbage had the highest antioxidant activity, followed by green cabbage, mustard cabbage, Chinese cabbage and Chinese white cabbage. In a study focused on *B. oleracea*, Podsedek *et al*. [23] found a comparable antioxidant activity between red cabbage and Brussels sprouts, which were from 5 to 2.2-fold higher than that for white and savoy cabbages. Authors suggest that the highest antioxidant activity of red cabbage compared to the other green and white cultivars may be due to the presence of different antioxidant components such as phenolic compounds.

Methods for identification and quantification of the main phenolic compounds have been generally based on HPLC coupled to DAD or MS detectors [13,46,47,48,49]. Polyphenol composition of members of the *Brassicaceae* family has been investigated. Nowadays, it is widely known that *Brassica* vegetables contain flavonoids, and especially flavonols. A large number of flavonoid glycosides have been found; among them, glycosides of kaempferol and quercetin, their derivatives in combination with hydroxycinnamic acids as well as sinapic acid derivatives have been found to be the most important phenolic compounds in *Brassica* species [18,50,51]. Most studies deal with *B. oleracea* crops like broccoli [52], cabbage [22,51] and kale [53]. In the last years, phenolic composition of other vegetable species like *B. rapa*, *B. napus* and *B. juncea* has also been studied [54,55,56]. In the following sections we will review the phenolic composition of the four vegetable species of the genus *Brassica* summarized in Table 2 and of other cruciferous crops from the family *Brassicaceae* in Table 3.

### 3.1. Phenolic composition in Brassica oleracea crops

Several studies have reported the presence of phenolic compounds in different *B. oleracea* crops [13,48,49,56,57]. Phenolic compounds content depends on the analysis method used as well as on numerous environmental factors. For this reason, phenolic content comparisons among crops evaluated under different conditions and with different analytical methods are not too accurate; therefore, this review mainly deals with the phenolic profile of *Brassica* crops, i.e. their qualitative differences rather than differences on concentrations.

Phenolics are distributed differently depending on the crop and on the plant part evaluated. External and internal leaves of different *B. oleracea* crops like tronchuda cabbage [58] and savoy cabbage [59] were found to be different in terms of total phenolic content. Quercetin, kaempferol and phenolic acids derivatives from the external and internal leaves, seeds and sprouts leaves of tronchuda cabbage have been reported by several authors [48,58,60,61] and the different composition seems to be determinant for the antioxidant activity displayed by each. Leaves contained higher amounts of phenolic compounds than stems and inflorescences. In another study, Sousa *et al*. [62] presented a similar qualitative phenolic composition in kales and tronchuda cabbage inflorescences, exhibiting several complex kaempferol derivatives and 3-*p*-coumaroylquinic acid (Table 2).

Among crops included into *B. oleracea* species, broccoli has been the most exhaustively studied with regard to polyphenol composition. Numerous recent studies have shown that this crop contains a high antioxidant potential linked to a high level of phenolic compounds [45,49,63] and it is a good source of flavonol and hydroxycinnamoyl derivatives. Llorach *et al*. [49] found 22 compounds that were identified as several derivatives of kaempferol and ferulic and sinapic acids. The two main flavonol glycosides present in broccoli florets are quercetin and kaempferol 3-*O*-sophoroside-7-*O*-glucoside and the other minor glucosides were isoquercitrin, kaempferol 3-*O*-glucoside and kaempferol 3-*O*-diglucoside [13,52] (Table 2). In the same way, more than 20 compounds were found in other *B. oleracea* crops such as kale (*B. oleracea* var. *acephala*), curly kale (*B. oleracea* var. *sabellica*), white cabbage (*B. oleracea* var. *capitata*), black cabbage (*B. oleracea* var. *acephala* DC. subvar. *viridis*), cauliflower (*B. oleracea* L. var. *botrytis*) and tronchuda cabbage (*B. oleracea* var. *costata*), where the main phenolics were kaempferol and quercetin 3-*O*-sophoroside-7*-O-*glucoside and its combinations with different hydroxycinnamic acids, mainly kaempferol and quercetin 3*-O-*(caffeoyl/sinapoyl)-sophoroside-7*-O-*glucoside [14,30,48,53,56,57,64] (Table 2). Heimler *et al*. [65] compared the main phenolics in several *B.* *oleracea* crops and reported that broccoli and kale varieties exhibit the highest content of both total phenolics and flavonoids. 

Ferreres *et al*. [66] and Taveira *et al*. [67] characterized the phenolic compounds and evaluated the antioxidant potential of shoots of *B. oleracea* var. *costata* grown *in vitro*, finding several compounds distinct from those described previously from material grown in the field. Authors detected a high number of chlorogenic acids, flavonoids (prevailing hydroxycinnamic acid esters of kaempferol and quercetin glycosides) and hydroxycinnamic acyl glycosides (with predominance of synapoyl gentiobiosides).The results obtained by these authors are relevant since they indicate that the *in vitro* production of shoots can become important as a dietary source of compounds with a health protective potential.

Anthocyanins have also been identified on *Brassica* vegetables [2,9,68]. For example, the red pigmentation of red cabbage and purple cauliflower is caused by anthocyanins. The major anthocyanins identified in cruciferous crops like red cabbage [68] or broccoli sprouts [9] are cyanidin 3-*O-*(sinapoyl)(feruloyl)diglucoside-5-*O-*glucoside and cyanidin 3-*O-*(sinapoyl)(sinapoyl)diglucoside-5-*O-*glucoside, with quantitative differences among species and crops within the species. For example, differences among cauliflower and red cabbage were found by Lo Scalzo *et al*. [12] in their anthocyanin profiles. Cyanidin-3,5-diglucoside was absent in cauliflower, while it was well represented in red cabbage. Anthocyanins in the genus *Brassica* present unusually complex structures with one or more cinnamic acids. The *p*-coumaryl and feruloyl esterified forms of cyanidin-3-sophoroside-5-glucoside were predominant in cauliflower, while the sinapoyl ester was mostly present in red cabbage [12].

In broccoli sprouts [9] qualitative and quantitative differences among varieties in the anthocyanin composition were found. These authors identified cyanidin-3-*O*-diglucoside-5-*O*-glucoside acylated and double acylated with *p*-coumaric, sinapic, caffeic, ferulic or sinapic acids with at least three predominant anthocyanins isomers of cyanidin 3-*O*-(acyl) diglucoside-5-*O*-glucoside, cyanidin 3-*O*-(acyl1)(acyl2) diglucoside-5-*O*-glucoside, and cyanidin 3-*O*-(acyl1)(acyl2) diglucoside-5-*O*-(malonyl) glucoside.

Significant levels of hydroxycinnamic acids have also been reported in *B. oleracea* species, like kale, cabbage, broccoli, and cauliflower. In these crops, hydroxycinnamoyl gentiobiosides and hydroxycinnamoylquinic acids were found to be the most abundant [13,69]. The predominant hydroxycinnamic acids conjugates have been identified as 3-caffeoyl quinic acid, 3-*p*-coumaroyl quinic acid, 1,2-disinapoylgentiobiose, 1-sinapoyl-2-feruloylgentiobiose, 1,2,2’-trisinapoylgentiobiose and 1,2´-disinapoyl-2-feruloylgentiobiose. More recently, Ayaz *et al*. [34] identified gallic, protocatechuic, *p*-hydroxybenzoic, vanillic, syringic, salicylic, *p*-coumaric, caffeic, ferulic and sinapic acids as the most abundant in kales. Significant levels of chlorogenic acids have previously been reported in leafy *Brassica* species, like kale, cabbage and Brussels sprouts. Inflorescences from kales, tronchuda cabbage and turnip tops exhibited the same six organic acids (aconitic, citric, pyruvic, malic, shikimic and fumaric acids), but kales presented a considerably higher amount [62].

**Table 2:** Variation of phenolic compounds found in main vegetable *Brassica* crops.

	*Brassica oleracea*					*Brassica rapa*		*Brassica napus*	*Brassica juncea*
Phenolic compound	Tronchuda cabbage^1^	Cauliflower^2^	Kale^3^	Broccoli^4^	White cabbage^5^	Turnip greens/tops^6^	Pak choi^7^	Leaf rape^8^	Leaf mustard^9^
**Quercetin (Q) derivatives**									
Q-3*-O-*sophorotrioside-7*-O-*sophoroside			x	X					
Q-3*-O-*sophorotrioside-7-glucoside			x	X		x			x
Q-3*-O-*sophoroside-7*-O-*glucoside	x	x	x	X	x				x
Q-3,7-di*-O-*glucoside			x	X	x	x		x	x
Q-3*-O-*sophoroside			x	X	x	x			x
Q-7*-O-*glucoside						x			
Q-3*-O-*glucoside	x			X		x			x
Q-3*-O-*(caffeoyl)-sophorotrioside-7*-O-*glucoside				X					x
Q- 3*-O-*(sinapoyl)-sophorotrioside-7*-O-*glucoside				X					x
Q-3*-O-*(feruloyl)-sophorotrioside-7*-O-*glucoside				X					x
Q-3*-O-*(*p*-coumaroyl)-sophorotrioside-7*-O-*glucoside				X					
Q-3*-O-*(caffeoyl)-sophoroside-7*-O-*glucoside			x	X	x	x		x	x
Q-3*-O-*(methoxycaffeoyl)-sophoroside-7*-O-*glucoside			x		x	x			
Q-3*-O-*(sinapoyl)-sophoroside-7*-O-*glucoside		x	x		x			x	x
Q-3*-O-*(feruloyl)-sophoroside-7*-O-*glucoside									x
Q-3*-O-*(*p*-coumaroyl)-sophoroside-7*-O-*glucoside				X					
Q-3*-O-*(feruloyl)-sophoroside			x	X	x	x		x	
**Kaempferol (K) derivatives**									
K-3*-O-*tetraglucoside-7*-O-*sophoroside	x								
K-3*-O-*sophorotrioside-7*-O-*sophoroside	x	x	x	X	x	x			
K-3*-O-*sohorotrioside-7*-O-*glucoside	x	x		X				x	x
K-3*-O-*sophoroside-7*-O-*diglucoside	x	x		X					x
K-3*-O-*sophoroside-7*-O-*glucoside	x	x	x	X	x	x	x	x	
K-3,7-di*-O-*glucoside			x	X	x	x	x	x	
K-3*-O-*sophoroside			x	X	x	x	x		
K-7*-O-*glucoside		x	x		x	x	x		x
K-3*-O-*glucoside	x			X					x
K-3*-O-*(caffeoyl)-sophorotrioside-7*-O-*sophoroside				X					
K-3*-O-*(methoxycaffeoyl)-sophorotrioside-7*-O-*sophoroside				X					
K*-O-*(sinapoyl)-sophorotrioside-7*-O-*sophoroside				X					
K*-O-*(feruloyl)-sophorotrioside-7*-O-*sophoroside				X					
K-3*-O-*(*p*-coumaroyl)-sophorotrioside-7*-O-*sophoroside				X					
K-3*-O-*(caffeoyl)-sophorotrioside-7*-O-*glucoside				X					x
K-3*-O-*(methoxycaffeoyl)-sophorotrioside-7*-O-*glucoside				X					
K*-O-*(sinapoyl)-sophorotrioside-7*-O-*glucoside				X					
K*-O-*(feruloyl)-sophorotrioside-7*-O-*glucoside				X					x
K-3*-O-*(caffeoyl)sophoroside-7*-O-*glucoside	x	x	x	X	x	x	x	x	x
K-3*-O-*(methoxycaffeoyl)sophoroside-7*-O-*glucoside	x		x		x	x	x	x	x
K-3*-O-*(sinapoyl)-sophoroside-7*-O-*glucoside	x	x	x		x	x	x	x	x
K-3*-O-*(feruloyl)-sophoroside-7*-O-*glucoside	x	x	x		x	x		x	x
K-3*-O-*(*p*-coumaroyl)-sophoroside-7*-O-*glucoside			x		x	x	x	x	
K-3*-O-*(methoxycaffeoyl)-sophoroside			x	X	x	x		x	
K-3*-O-*(sinapoyl)-sophoroside	x		x		x	x		x	
K-3*-O-*(feruloyl)-sophorotrioside	x								
K-3*-O-*(feruloyl)-sophoroside	x		x		x	x		x	
K-3*-O-*(*p*-coumaroyl)-sophoroside			x		x	x			
**Isorhamnetin (I) derivatives**									
I-3-sophorotrioside-7-sophoroside									
I-3,7-di*-O-*glucoside						x	x		x
I-3-glucoside						x	x		x
**Hydroxycinnamic acids**									
3-caffeoyl quinic acid		x	x		x	x	x	x	x
5-caffeoyl quinic acid									x
3-*p*-coumaroyl quinic acid	x		x		x	x		x	x
4-*p*-coumaroyl quinic acid	x								x
4-caffeoyl quinic acid			x		x				
Sinapylglucoside			x		x	x		x	
Ferulic acid			x			x	x	x	x
4-feruloyl quinic acid			x		x				x
Sinapic acid	x		x		x	x	x	x	x
1,2-disinapoylgentiobiose	x	x	x	X	x	x	x	x	x
1-sinapoyl-2-feruloylgentiobiose	x	x	x	X	x	x	x	x	x
1,2,2’-trisinapoylgentiobiose	x	x	x	X	x	x	x	x	x
1,2’-disinapoyl-2-feruloylgentiobiose	x	x	x	X	x	x	x	x	x

^1^Tronchuda: [48,62]; ^2^Broccoli: [13,45,49,52,63]; ^3^Cauliflower: [64]; ^4^White cabbage: [56,57,59]; ^5^Kale: [14,56]; ^6^Turnip greens: [55,72]; ^7^Pak choi: [47]; ^8^Leaf rape: [56]; ^9^Leaf mustard: [15,70]

**Table 3:** Phenolic compounds in different cruciferous crops.

Crop	Compound	Reference
*Moricandia arvensis*	3,4’-di*-O-*β-D-glucopyranoside-7*-O-*α-L-rhamnopyranoside	[80]
	β-D-glucopyranosyl 4*-O-* β-D-glucopyranosylcaffeate	
	methyl 3*-O-* β-D-glucopyranosyl-5-hydroxycinnamate	
	β-D-glucopyranosyl 4*-O-*β-D-glucopyranosylbenzoate	
	β-D-glucopyranosyl 4-hydroxybenzoate	
	methyl 4*-O-*β-D-glucopyranosylcaffeate	
	1*-O-*caffeoyl-β-D-glucopyranoside	
	2-phenylethyl-β-D-glucopyranoside	
*Bunias orientalis*	Kaempferol monosinapoyl di-*O*-glycoside	[82]
	Kaempferol monomalonyl/monosinapoyl di-*O*-glycoside	
	Kaempferol di*-O-*glucoside and tri*-O-*glucoside	
	demethylated sinapic acid	
*Diplotaxis erucoides/Eruca sativa*	Kaempferol di*-O-*glycoside	[82]
	Isorhamnetin mono*-O-*,di*-O-*, and tri*-O-*glycosides	
	Quercetin monosinapoyl tri*-O-*glycoside	
	Quercetin tri*-O-*glycoside	
	Quercetin tetra*-O-*glycoside	
	Quercetin monosinapoyl di*-O-*glycoside	
	Quercetin di*-O-*, tri*-O-*, and tetra*-O-*glycosides	
*Diplotaxis tenuifolia*	Quercetin-3,3‘,4‘-triglucoside	[83]
	Quercetin-3,4‘-di-glucoside-3‘-(6-methoxycaffeoyl-glucoside)	
	Quercetin-3,4‘-di-glucoside-3‘-(6-caffeoyl-glucoside)	
	Quercetin-3,4‘-di-glucoside-3‘-(6-sinapoyl-glucoside)	
	Quercetin-3,4‘-di-glucoside-3‘-(6-feruloyl-glucoside)	
	Quercetin-3,4‘-di-glucoside-3‘-(6-*p-*coumaroyl-glucoside)	
	Quercetin-3-(2-methoxycaffeoyl-glucoside)-3‘-(6-sinapoyl-glucoside)-4‘-glucoside	
	Quercetin-3-(2-caffeoyl-glucoside)-3‘-(6-sinapoyl-glucoside)-4‘-glucoside	
	Quercetin-3-(2-sinapoyl-glucoside)-3‘-(6-sinapoyl-glucoside)-4‘-glucoside Quercetin-3-(2-feruloyl-glucoside)-3‘-(6-sinapoyl-glucoside)-4‘-glucoside	
	Quercetin-3-(2-feruloyl-glucoside)-3‘-(6-feruloyl-glucoside)-4‘-glucoside	
	Kaempferol-3,4‘-di-glucoside	
	Isorhamnetin-3,4‘-di-glucoside	
*Eruca vesicaria*	Quercetin-3-glucoside	[83]
	Kaempferol-3-glucoside	
	Kaempferol-3,4‘-di-glucoside	
	Kaempferol-3-(2-sinapoyl-glucoside)-4‘-glucoside	
	Isorhamnetin-3-glucoside	
	Isorhamnetin-3,4‘-di-glucoside	
*Nasturtium officinale*	Quercetin 3*-O-*triglucoside-7*-O-*rhamnoside	[50]
	Quercetin 3*-O-*gentiobioside-7*-O-*rhamnoside	
	Quercetin 3*-O-*sophoroside-7*-O-*rhamnoside	
	Quercetin 3*-O-*sophoroside-7*-O-*(caffeoyl)-rhamnoside	
	Quercetin 3*-O-*triglucoside-7*-O-*(caffeoyl)-rhamnoside	
	Quercetin 3*-O-*triglucoside-7*-O-*(sinapoyl)-rhamnoside	
	Quercetin 3*-O-*triglucoside-7*-O-*(feruloyl)-rhamnoside	
	Kaempferol 3*-O-*triglucoside	
	Kaempferol 3*-O-*sophoroside	
	Kaempferol 3*-O-*gentiobioside	
	Kaempferol 3*-O-*gentiobioside-7*-O-*rhamnoside	
	Kaempferol 3*-O-*triglucoside-7*-O-*rhamnoside	
	Kaempferol 3*-O-*triglucoside-7*-O-*(caffeoyl)-rhamnoside	
	Kaempferol 3*-O-*triglucoside-7*-O-*(sinapoyl)-rhamnoside	
	Kaempferol 3*-O-*triglucoside-7*-O-*(sinapoyl)-rhamnoside	
	Kaempferol 3*-O-*sophoroside-7*-O-*rhamnoside	
	Kaempferol 3*-O-*sophoroside-7*-O-*(caffeoyl)-rhamnoside	

Hydroxycinnamic acids in leaves and stems of tronchuda cabbage varieties were also evaluated by Ferreres *et al*. [58]. In this work, authors found that hydroxycinnamoyl gentiobiosides of ferulic and sinapic acid were predominant in the inner leaves of tronchuda cabbage, while hydroxycinnamoyl-quinic acids were more abundant in leaves and stems. 

### 3.2. Phenolic composition in Brassica rapa crops

In contrast to *B. oleracea* vegetables, and despite being much appreciated and highly consumed, phenolic composition of *B. rapa* crops has been less investigated. However, this subject has received an increasing interest in the last years. At present, it is known that crops from this species contain a high amount of phenolic compounds and as result, they are an appreciable source of polyphenols, especially flavonoids. The main polyphenols identified in *B. rapa* vegetables are acylated mono-, di-, tri- and tetraglucosides of quercetin, kaempferol and isorhamnetin as well as esters of hydroxycinnamic acids with malic acid, glycosides, and quinic acid [47,70]. In fact, the main difference between *B. oleracea* and *B. rapa* species is the presence of isorhamnetin derivatives in the *B. rapa* group, which are always absent in *B. oleracea* [71]. 

Ferreres *et al*. [72] and Francisco *et al*. [55] identified more than 20 acylated and nonacylated flavonol glycosides and ferulic and sinapic acids in *Brassica rapa* var. *rapa* by HPL-DAD-ESI-MSn (Table 2). The content of phenolic compounds was studied in turnip edible parts including leaves, stems, flower buds and roots [54,55]. The authors found that kaempferol 3-*O*-sophoroside-7-*O*-glucoside, kaempferol 3-*O*-(feruloyl/caffeoyl)-sophoroside-7-*O*-glucoside, kaempferol 3,7-di-*O*-glucoside, isorhamnetin 3,7-*O*-diglucoside and sinapic acid were the major phenolics quantified by HPLC–DAD analysis. In a comparative study carried out by Sousa *et al*. [62] in *Brassica* inflorescences, it has been shown that *B. rapa* exhibited several phenolic acids and flavonoids distinct from those found in the *B. oleracea* species, mainly isorhamnetin derivatives. In another *B. rapa* variety, namely var. *sylvestris,* Romani *et al*. [71] also found some common compounds described in *Brassica rapa* var. *rapa* such as derivatives of kaempferol 3,7-di-*O*-glucoside, kaempferol-3-*O*-glucoside, isorhamnetin 3,7-*O*-diglucoside and hydroxycinnamoyl gentiobiosides, being kaempferol-3-*O*-glucoside and quercetin-3-*O*-(sinapoyl)-sophotrioside-7-*O*-glucoside the most abundant compounds.

In a recent study, the phenolic profiles of fifteen *B. rapa* crops, including *B. rapa* var. *pekinensis*, *B. rapa* var. *chinensis*, *B. rapa* var. *oleifera*, *B. rapa* var. *ruvo* and *B. rapa* L. var. *rapa* were reported by Lin and Harnley [15]. Those groups included vegetable crops which are among the major *Brassica* vegetables consumed in China and some Asian countries*.* The major phenolic compounds identified were kaempferol 3-*O*-sophoroside-7-*O*-glucoside derivatives, isorhamnetin 3-*O*-glucoside-7-*O*-glucoside, hydroxycinnamoyl gentiobioses, hydroxycinnamoylmalic acids and hydroxycinnamoyl-quinic acids. The phenolic compounds identified on these Asian *Brassica* vegetables were similar to those of the abovementioned European *B. rapa*.

Turnip roots seem to be a less interesting edible part compared to leaves or inflorescences due to the very low amounts of phenolic compounds and the small antioxidant capacity found in this organ. In a study performed by Fernandes *et al*. [54], only ferulic and sinapic acids and their derivatives were detected in significant amounts in this organ.

### 3.3. Phenolic composition in Brassica napus crops

Most efforts in this species have been focused on crops like rapeseed (*Brassica napus* var. *oleifera*). It is known that seeds of winter rapeseed varieties contain high amounts of phenolic compounds. In fact, canola seeds are much richer in phenolic compounds compared to other oilseeds. The most significant phenolic compounds are sinapic acid derivatives [73,74], although other minor phenolics in rapeseed include *p*-hydroxybenzoic, vanillic, gentisic, protocatechuic, syringic, *p*-coumaric, ferulic, caffeic and chlorogenic acids. In leaves of oilseed rape, four hydroxycinnamic acids (caffeic, *p*-coumaric, ferulic and sinapic acid) were identified in the water-soluble phenolic fraction of the leaves. 

Although *B. napus* crops are mainly used as oilseed, this species also include forage and vegetable types likes rutabaga or swede and leaf rape or nabicol (Table 2). Contrary to other *Brassica* vegetables, few studies have been done to evaluate the phenolic profile of vegetable *B. napus*. Velasco *et al*. [56] identified phenolics present in nabicol leaves, which is a vegetable *Brassica* crop widely grown in the Northwest of Spain. They identified 17 flavonoids, mostly derivatives from kaempferol and eight hydroxycinnamic acids, being sinapic acid the most abundant (Table 2). Li *et al*. [75] generated transgenic *B. napus* plants over expressing the Arabidopsis PAP1 *(AtPAP1)* gene responsible for the production of the anthocyanin pigment 1 and identified and quantified the leaf phenolics in transgenic plants and non-transgenic controls. They identified derivatives of quercetin, kaempferol, sinapic acid, cyanidin and pelargonidin and also found that all of them, except for the kaempferol derivatives were dramatically increased in the leaves of transgenic plants as compared to the non-transgenic controls.

### 3.4. Phenolic composition in Brassica juncea crops

*Brassica juncea* is mainly used as a condiment because of its seeds, along with the other mustards of the genus *Brassica*, *Brassica carinata* and *Brassica nigra*. Nevertheless, *B. juncea* leaves are also consumed as vegetables in Asian countries (Table 1). The phenolic composition of this species has been less studied with regard to *B. oleracea* and *B. rapa.* The main flavonols in this species are quercetin and kaempferol. The phenolic compound variation of two Chinese leaf mustard cultivars grown under field conditions was studied by HPLC–ESI–MSn analysis [70,76]. Authors identified the free polyphenol content in the outer and inner leaves as well as in their leaf blades and leaf stalks, and concluded that hydroxycinnamic acids and flavonoids were higher in the leaf blade than in the stalk, whereas small amounts of flavonoids were detected in the stalks. The main flavonoids are kaempferol derivatives (mono-, di-, triglucosides). Isorhamnetin and hydroxycinnamoyl gentiobioses were also detected, but no quercetin derivatives. The main hydroxycinnamic acids were malate derivatives of sinapic, ferulic, hydroxyferulic and caffeic acids. Ferulic acid content was significantly higher in the leaf blade than in the stalk.

Fang *et al*. [77] determined the contents of the total free phenolic acids, the total phenolic acids, the total phenolics and the antioxidant activities in leaf mustard as well as the effects of pickling methods on these compounds and they identified several hydroxycinnamic acids as caffeic, *p*-coumaric, ferulic and sinapic along with benzoic acid derivatives as gallic, protocatechuic, *p*-hydroxybenzoic, and vanillic acids.

### 3.5. Phenolic composition in other cruciferous crops

Besides vegetable *Brassica* species belonging to the U triangle [78], *Brassicaceae* is a large family of plants that include important vegetable crops. Several species within the family *Brassicaceae* have been surveyed for their flavonoid profiles. In these studies, flavonol glycosides were the only flavonoids present in leaves and flowers of the genera *Brassica* and *Sinapis*. In a chemosystematic survey on wild *Brassica* relatives, Aguinagalde *et al*. [79] identified 21 different flavonoid glycosides, all based on the flavonol skeleton. Braham *et al*. [80] identified phenolic compounds in *Moricandia arvensis* (Table 3) and evaluated their antioxidant capacity, finding that some of the compounds detected possess a high scavenging activity.

Flavonols were also reported in *Diplotaxis* and *Eruca* leaves by Weckerle *et al*. [81]. More recently, Bennet *et al*. [82] studied the phenolic compound composition in several cruciferous species such as *Diplotaxis erucoides* L., *Diplotaxis tenuifolia* L., *Eruca sati*v*a* L., and *Bunias orientalis* L., all of them known as rocket crops. These crops had significant levels of polyglycosylated flavonoids, with/without hydroxycinnamoyl acylation and the most abundant flavonoids were kaempferol, quercetin and isorhamnetin. This composition was found in all tissues, except roots. Hydroxycinnamate derivatives of either the disaccharide gentiobiose or the quinic acid were detected at very low levels, in leaf and floral tissues of *Bunias*, *Diplotaxis* and *Eruca* species (Table 3). Martinez-Sanchez *et al*. [83] also studied the flavonoid profile of *Eruca vesicaria* and *D. tenuifolia* (Table 3). They found important differences between flavonoid profiles of these two species. *E. vesicaria* contained kaempferol derivatives as principal compounds, whereas *D. tenuifolia* instead accumulated quercetin derivatives. Some diacyl derivatives found in these species have not been identified in other *Brassicaceae.* Like other *Brassicaceae*, the flavonoids found in watercress (*Nasturtium officinale*) were quercetin and kaempferol derivatives glycosylated and acylated, but unlike them, they showed a characteristic glycosylation pattern with rhamnose at the 7 position [50] (Table 3).

In another study, Onyilagha *et al*. [84] presented a comprehensive review of flavonoid distribution in different tissues of crops from the family *Brassicaceae*. Authors also studied the leaf flavonoids in several cruciferous species, including *Sinapis alba, Thlaspi arvense*, *Camelina sativa*, *Crambe* spp. and several other genera of the family *Brassicaceae*. These authors reported the accumulation of derivatives of flavonols, quercetin, kaempferol and isorhamnetin in *S. alba*; quercetin, in *C. sativa;* quercetin and kaempferol in *C. hispanica* var. *glabrata* and derivatives of the flavones, apigenin and luteolin, in *C. abyssinica, C. hispanica* var. *hispanica* and *T. arvense* leaves. Various kaempferol, quercetin, and isorhamnetin glycosides were identified in the leaves of flowering *Diplotaxis* species. In leaves of *B. orientalis*, various hydroxycinnamate derivatives, including sinapoylglucose were found; no data on flavonoids were reported. Quercetin triglucosides were found in *E. sativa* by qualitative analyses by liquid chromatography/mass spectrometry (LC/MS) and NMR.

## 4. Variation on Phenolic Content in *Brassica* Vegetables

Biosynthesis and concentration of phenolic compounds in plants depends on genetic and environmental factors. Several studies have demonstrated that there is a substantial and significant variation for the antioxidant phytochemicals into *Brassica* species, both within and among species, and even among crops of the same species; thus, the potential health benefits provided by cruciferous crops will depend firstly on the genotype. The phenolic compound composition may differ between cultivars, as well as among parts within the individual plant as shown in several crops like turnip greens and turnip tops [85], pak choi [47] and tronchuda cabbage [54,58]. These compounds are also susceptible to ontogenic variation but the published studies that have addressed this topic have been inconsistent. 

On the other hand, secondary metabolites present in *Brassica* crops are very susceptible to changes in environmental conditions. Phenolic contents are affected by biotic stresses (insect attack and pathogen infection) and abiotic stresses (light, temperature, nutrient supplies, water availability, growing conditions and UV radiation) besides storage conditions, post-harvest treatments and the estimation methods [86,87]. All these factors, besides the biosynthesis of phenolic antioxidant compounds, affect the final concentration of polyphenols in plant tissues. As it was previously explained, phenolics are produced in plants as secondary metabolites via the shikimic acid pathway. Phenylalanine ammonialyase (PAL), the key enzyme catalyzing the biosynthesis of phenolics from the aromatic amino acid phenylalanine, was found to be responsive to biotic and abiotic stresses. 

In the following sections some factors like fertilization, processing, cooking methods or storage conditions are discussed in detail.

### 4.1. Influence of fertilization and cropping systems on phenolic content

A crucial question about *Brassica* vegetables that deserves full attention in scientific literature is the edaphic conditions in which they are grown. Plant quality can be modified by growing plants in a high mineral-containing medium, thus attaining high levels of nutritionally important minerals that can be used to produce foods and food ingredients dietary supplements. Plant nutrients can be thus important factors in determining the secondary metabolism synthesis within plants. Nitrogen is one of the most important essential plant nutrients in controlling quality and yield of vegetables. Moreover, nitrogen modulates the biosynthesis of secondary metabolites (e.g., phenolic compounds, glucosinolate, carotenoid, *etc.*). Many plant species, particularly *Brassicaceae* crops, incorporate sulfur into a wide range of secondary compounds such as the sulfation of flavonol and desulfoglucosinolates, choline and gallic acid glucoside. Sulfur is an essential plant macronutrient found in cysteine and methionine amino acids, as well as in a variety of secondary metabolites. However, few studies have investigated the impact of nitrogen and sulfur application on the total phenolic concentrations and antioxidant activity. 

Several studies reported that an increase in sulfur fertilization significantly promotes the total phenolic contents and the antioxidant activity in leaf mustard [88], broccoli [89] and ‘friarello’, a local *B. rapa* crop widely grown in Southern Italy [87]. Li *et al*. [88] also determined the effect of nitrogen supply on leaf mustard and found that the total phenolic content was considerably decreased by increasing nitrogen fertilization. These studies provide clear evidence that nitrogen and sulfur nutrition can be used to manipulate total phenolic concentrations of *Brassica* crops with potential benefits to human health and as a result, it can be concluded that sulphur fertilization may improve the nutritional value of these crops.

Like other secondary metabolites, phenolic compounds may be affected by agronomic practices. Several studies have compared the contents of certain phytochemicals between organic and conventional fruits and vegetables [60,90,91]. Sousa *et al*. [60] studied the content of phenolic compounds in tronchuda cabbage under organic and conventional agriculture and they conclude that generally, leaves from organic culture have higher amounts of phenolics, probably due to the interference of mineral fertilizers and pesticides with the biosynthetic pathway of phenolic compounds. In another study, Young *et al*. [91] found that pak choi samples grown organically had higher levels of total phenolics than conventional samples, but these differences among organic and conventional growing were not found in collards. They concluded that the production method did not increase the biosynthesis of phenolics but the organic system provided an increased opportunity for insect attack, resulting in a higher level of total phenolic agents in pak choi. Thus, insect attack might be a biotic stress factor contributing to higher levels of total phenolic agents in some vegetables from organic production systems.

### 4.2. Influence of processing and cooking on phenolic composition

Phenolics in vegetables exist in both free and conjugated forms. Generally, only conjugated flavonoids are present in fresh vegetables, but aglycones may be found as a result of food processing. It is known that processing may affect the concentration and biological activities of different compounds present in plants to a significant extent. This aspect seems to be very important taking into account that only some vegetables are consumed in a raw state and most of them are processed before consumption. The recent literature data show a consistent trend for the effects of thermal processing on the total antioxidant activity in vegetable *Brassica* crops when comparing to other vegetable crops; however, when compare to the total flavonoid or total phenolic content, the results did not show such consistency. This suggests that the effect of thermal processing on phenolic, flavonoid or total antioxidant activities is different in different products and deserve further research. In addition, differences in processing methods may have different effects on the content of distinct phytochemicals. Industrial processing such as blanching, canning, sterilizing and freezing, as well as cooking methods are expected to affect the yield, chemical composition and bioavailability of antioxidants [92]. *Brassica* vegetables containing phenolic compounds usually undergo domestic processing before cooking. Some of them such as broccoli or cauliflower are cut; others such as kale and cabbage are prepared in julienne, which may have similar effects to chopping. Operations such as cutting and slicing may cause a rapid enzymatic depletion of several naturally occurring antioxidants as a result of the cellular disruption, which allows contacts of substrates and enzymes. During vegetable cooking, qualitative changes, antioxidant breakdown and their leaching into surrounding water may influence the antioxidant activity of the vegetables [1]. 

Some antioxidant compounds like ascorbic acid and carotenoids are very sensitive to heat and storage and are lost during different vegetable processing steps [93]. However, flavonoids and some phenolic compounds are quite stable at high temperature and over long periods of storage [94]. Several studies have shown that blanching has a significant effect on the contents of ascorbic acid and total phenolics, and on the antioxidant activity of green leafy vegetables. Blanching of vegetables does not necessarily cause the loss of antioxidant properties. In some vegetables, blanching might actually increase the availability of the natural occurring antioxidant components besides improving the palatability of vegetable crops.

The literature data have shown that the loss of dietary antioxidants is caused by the cooking conditions, such as the type of cooking (conventional, steaming, microwaving, *etc.*), cooking time and amount of water. A loss of antioxidant capacity after boiling has been observed for several vegetables [93]. It is well known that the cooking process drastically reduces the vitamin C content of vegetables and several other authors report a loss in the phenolic content of vegetables after cooking. The overall loss of antioxidants (for oxidation, as in the case of vitamin C or for a simple diffusion in the cooking water, as in the case of phenolics) results in the decrease of antioxidant capacity. In another study, Vallejo *et al*. [94] compared the losses in phenolics compounds when broccoli was submitted to high-pressure boiling, low-pressure boiling, steaming and microwaving. The authors found clear disadvantages when microwave cooking was used, noticing losses of 97, 74 and 87% in flavonoids, sinapic acid derivatives and caffeoylquinic acid derivatives, respectively. To this respect, Zhang and Hamauzu [93] reported losses in the total phenolics of 62% in broccoli florets and of 43% in broccoli stems. However, they did not find differences with traditional boiling, which suggests that, as no soaking effect is produced, losses may be attributed to degradation. Similar results were found by Francisco *et al*. [85] who reported a loss of 65–75% of flavonoids and 70–80% of hydroxycinnamic acids under conventional and high-pressure cooking in turnip tops. Nevertheless, losses were reduced to 20–30% by steaming cooking, showing that this is the ideal method to preserve secondary metabolites in *Brassica* crops.

Lin and Chang [95] examined the antioxidant activity of broccoli under different cooking treatments and found that a precooking and/or cooking treatment had no profound effect on the antioxidant properties of broccoli. In another study, Sultana *et al*. [96] reported the effects of different cooking methods (boiling, frying and microwave cooking) on the antioxidant activity of some selected vegetables including cabbage, cauliflower, yellow turnip and white turnip and concluded that all the cooking methods affected the antioxidant properties of these vegetables; however, microwave treatment exhibited more deleterious effects when compared to those of other treatments. Most phenolic compounds are water soluble and they are recovered in the water after cooking [97]. Authors found that the steam-cooking of broccoli results in an increase in the content of flavonoids and phenolic acids as compared to fresh broccoli, whereas cooking in water has the opposite effect. The increase in the content of polyphenols and carotenoids is related to their enhanced availability whereas the observed losses of the compounds are mainly due to their leaching into the cooking-water. 

However, other studies reported very slight losses of total flavonoids and caffeoylquinic derivatives in broccoli (11% and 8% respectively), while no loss of total sinapic and feruloyl derivatives occurred [94]. During steaming, phenolic compounds can remain in the edible part of broccoli, probably owing to the inactivation of oxidative enzymes [94]. Natella *et al*. [98] concluded that microwave and pressure cooking are less detrimental than boiling to the phenolics content of several vegetables, including cauliflower. As a conclusion, and because data in this sense are still ambiguous, moderate blanching time, proper handling, and an appropriate method might be sought for the processing of vegetable *Brassica* crops in order to preserve their antioxidant properties.

### 4.3. Influence of storage on phenolic content

Several studies indicate an increasing content of polyphenols for material stored under different modified atmosphere packaging for few days at low temperature. This might be an indication of further biosynthesis of polyphenols for plant protection in the first days after harvest [99], presumably triggered as a reaction to stress in the plants. Furthermore, it has been reported that longer storage times resulted in a decreased phenolic content, e.g. for flavonol glucosides in several vegetable crops [16]. In *Brassica* crops, the influence of storage on free phenolic content was recently studied in different cultivars of Chinese cabbage cultivated in Germany under field conditions [70]. These authors found that storage at 20 ºC resulted in rapid yellowing and floppy leaves which resulted in an undesirable appearance and sensory quality for consumers. The increasing levels of polyphenols observed in different works in the plant from post-harvest treatments (storage) open up possibilities for increased phenolic content in vegetables and foods.

## 5. Future Perspectives

Given the mounting data in support of the role of phenolics in the prevention of different chronic diseases including several types of cancer, improving the phenolic load of plant-origin-foods would be of a clear benefit to human health through dietary intervention. Plant geneticists have attempted to improve phytochemical levels through traditional breeding programs or through bioengineering of Phenylalanine ammonialyase (PAL) leading to secondary metabolite accumulation. To our knowledge, breeding programs to increase or decrease the content of a particular phenolic compound related to human health with horticultural *Brassica* crops have not been carried out. However, the modification of the synthesis of phenolic compounds is being currently carried out in different crops and in the model species *Arabidopsis thaliana*, to reduce or to increase the final concentration of a certain type of phenolic compound. 

To date, most of the structural and several regulatory genes of the synthesis pathways of phenolic compounds have been cloned, characterized and used in gene transformation experiments to modify their content. Respecting flavonoids pathway, early attempts to manipulate flavonoid biosynthesis were made to generate novel flower colors, such as the use of a maize DFR gene to produce a new flower colors in Petunia [100]. The use of structural genes in the metabolic engineering of flavonoids becomes more important when attempting to direct flavonoid synthesis towards branches that are normally absent in the host plant. This approach was used by Jung *et al*. [101], who introduced the IFS gene into *Arabidopsis* in order to convert naringenin, which is ubiquitous in higher plants, to the isoflavone genistein. An example of modification of transcriptional factors can be found in Schijlen *et al*. [102]. They introduced the transcriptional factors LC and C1 from maize into *Arabidopsis* and tobacco and this resulted in an accumulation of anthocyanins in tissues where they are not normally synthesized. 

With regard to the modification of the hydroxycinnamates pathway, an example can be found in *B. napus*. Seeds of oilseed rape accumulate high amounts of antinutritive sinapate esters [103]. Together with the high fiber content, the sinapate esters contribute to the antinutritive characteristics of the *B. napus* seed protein fraction as revealed by their bitter taste, astringency and low digestibility [104]. Therefore, reduction of the amount of sinapate esters in the seeds is a major goal in *B. napus* breeding. So far, conventional plant breeding has not been able to produce oilseed rape lines with a low-sinapate ester trait. Several studies reported on a large genetic variability of sinapate ester content and composition in seeds [105]. Analysis of variance, however, showed a highly significant effect of the environment as well. Thus, the transgenic approaches provided so far the best strategy to follow to finally obtain *B. napus* cultivar with low content in sinapate esters.

Following this research line, and based on the absence of soluble sinapate esters in the *A. thaliana* mutant *sin1*, which is impaired in ferulate-5-hydroxylase (F5H), the homologous gene from *B. napus* was identified and the sequence information used for an antisense suppression strategy [106]. This approach resulted in transgenic plants displaying a seed sinapine content reduced to 40% compared to control lines. Transgenic lines of *B. napus* were homozygous for a single insertion of a dsRNAi cassette designed to suppress the BnSGT1 gene in seeds. These lines showed a sinapate ester content of only about 30% relative to control plants [103]. Bhinu *et al*. [107] based in knowledge gained from *Arabidopsis*, developed transgenic *B. napus* lines which showed up to 90% reduction in sinapine. Information gains from these and other experiments carried out in other species will greatly contribute to a better undestanding of phenolic compounds synthesis and may be useful for *Brassica* crop improvement.

On the other hand, and it was previously explained, the flavonoid content is quite high in some *Brassica* species. For this reason, consumption of *Brassica* vegetables is highly recommended. However, a question to resolve nowadays is that of the adequate intake of these beneficial vegetables since as it has been noted by Jahangir *et al*. [2], the potentially toxic effects of excessive flavonoid intake are still largely ignored. At high doses, flavonoids may act as mutagens and, therefore, their unfavorable effects may well balance up their beneficial ones. Further research must be led about the toxicological properties of flavonoids, thus, clarifying the balance of potentially adverse and beneficial effects included in their mechanisms of action. 

It can be concluded that vegetables belonging to the family *Brassicaceae* are rich food sources of natural antioxidants and essential nutrients (vitamins, phenolics, minerals, fibre, *etc.*) and the vegetables of this family possess a high potential to manage against oxidative stress and, thus, act as strong anticancerous as well as antidegenerative foods. Therefore, for improving the quality and production of these vegetables, breeding programs are necessary in order to enhance the antioxidant potential of our daily food supply. Therefore, the potential of these phytochemical compounds for the maintenance of health and protection against heart disease and cancer is also raising interest among scientists and food manufacturers as consumers move towards functional foods with specific health effects. An interesting aspect for future research is to clarify the genotype × environmental interactions on the flavonoid composition in plants. By combining the knowledge gained from the studies concerning the effects of different flavonoid compounds on human health, it might be possible to produce plants with even better health properties. 

Understanding the bioavailability, transport and metabolism of polyphenols after consumption of *Brassica* vegetables as food is a prerequisite for understanding the mechanisms of their protective effects in humans. Research is also needed on the bioavailability and metabolism of polyphenols to allow scientific backed statements and recommendations on dietary intake, effective dosage, daily allowance and dietary guidelines for nutrition and health applications. Epidemiological and intervention studies examining the effects of phenolics in humans and animal models with relevant and reliable biomarkers of safety, ingestion, metabolism and functional activity might be improved to optimize phenolic bioavailability and realize their chemopreventive and chemotherapeutic effects in vivo. Furthermore, a better understanding of the dietary phenolic and gut microbiota relationship should help in the prevention of diseases as well as in improvement of human health.
